# Evaluating the effectiveness of the CAIDS-Q for screening for intellectual disability in mainstream schools

**DOI:** 10.1080/20473869.2024.2410524

**Published:** 2024-10-03

**Authors:** Karen McKenzie, George Murray, Dale Metcalfe, Simon Hackett

**Affiliations:** a Department of Psychology, Northumbria University, Newcastle upon Tyne, UK; b c/o NEAT Academy Trust, North Shield, UK; c Population Health Sciences Institute, Faculty of Medical Sciences, Newcastle University, Newcastle upon Tyne, UK

**Keywords:** Intellectual disability, CAIDS-q, schools, identification, Child and Adolescent Intellectual Disability Screening Questionnaire, education, psychometric properties, screening

## Abstract

**Objectives:**

The identification of intellectual disability in children can help them access targeted support and interventions. Research suggests that current methods of identifying intellectual disability in schools in the United Kingdom need to be improved. Screening tools can play a role in this. The study examined the performance of the Child and Adolescent Intellectual Disability Screening Questionnaire (CAIDS-Q) in mainstream schools as a means of facilitating identification of intellectual disability.

**Methods:**

Parents and/or teachers of 70 children completed the CAIDS-Q and an assessment of adaptive functioning about them. Children who were indicated as having difficulties completed an intellectual assessment. The data were used to explore some psychometric properties of the CAIDS-Q.

**Results:**

The CAIDS-Q had sensitivity and specificity values of 100% and 79% respectively when completed by teachers and 92% and 70% when completed by parents. CAIDS-Q scores correlated positively with intellectual and adaptive functioning scores, with large and medium effect sizes respectively. There was moderate agreement between parents and teachers on the outcome of the CAIDS-Q.

**Conclusions:**

The CAIDS-Q, when used in mainstream schools, had psychometric properties consistent with those found in health service settings. It may offer a way of improving identification of intellectual disability in educational settings.

## Introduction

To meet the criteria for intellectual disability, a person must experience lifelong, significant, global challenges with their intellectual functioning and daily living skills, with onset during childhood (World Health Organization 2022). These criteria highlight that people with an intellectual disability will require support in their day to day lives, the extent of which will depend on the severity of their intellectual disability. While support can be provided without the need for formal diagnosis, identifying that someone has an intellectual disability can help them to access more specialist services or targeted interventions (American Psychiatric Association 2024).

In the United Kingdom (UK), the use of different terminology to describe intellectual disability, such as ‘learning disability’ (Cluley 2018), as well as the inclusion of children with an intellectual disability within broader categories, such as those with special educational needs and disabilities (Department for Education and Department of Health 2015), can make it difficult to specifically identify the prevalence and needs of those with an intellectual disability.

Research also suggests that current systems in the UK which are used to identify children who have such additional educational support needs, including those with an intellectual disability, are inadequate. A recent analysis found that the main predictor of being identified was the primary school that the child attended rather than their individual characteristics (Hutchinson 2021). In this context, it is perhaps unsurprising that the identification of intellectual disability in children can be challenging, especially for those whose difficulties are milder (Delahunty et al. 2022). Many experience long delays in having their intellectual disability recognised, while for some, it may be missed completely (McKenzie et al. 2019a).

Identifying those who have an intellectual disability is important for many reasons. At population levels, it can help inform public health strategy and initiatives (Friedman, Parrish, and Fox 2018). At the individual level, it can help ensure that the child receives appropriate support. For example, in addition to the challenges that they face in relation to their cognitive and adaptive functioning, children with an intellectual disability may also experience associated psychosocial challenges, such as stigma, low self-esteem (McKenzie, Murray, and Derries 2020; Scior et al. 2020), and persistent health inequities (Kings College London 2023) that can further undermine their functioning and quality of life. Early identification can increase understanding of the child, guide interventions and identify support needs (see McKenzie et al. 2021). Early intervention can improve the social, adaptive, and cognitive functioning of the child (Guralnick 2017), help address difficulties with behaviour and sleep and improve family functioning (see Sapiets, Totsika, and Hastings 2021).

Accurate identification is also important because different conditions, such as intellectual disability and autism, may commonly co-occur and have aspects of their presentation that overlap (Thurm et al. 2019). It is important to differentiate between the two conditions because the experiences, support needs, and clinical outcomes are likely to differ depending on whether the child has an intellectual disability, autism, or both (see McKenzie, Metcalfe, and Murray 2023 for an overview).

In order to access early intervention, there first needs to be a recognition of potential need, followed by formal identification of need. Raising awareness amongst staff who work with children with conditions such as intellectual disability is highlighted as one way of facilitating this process (Sapiets, Totsika, and Hastings 2021). Teachers are well positioned to pick up early indicators that suggest children are likely to have an intellectual disability and the growing emphasis on inclusive education means that teachers in mainstream schools are increasingly likely to encounter children with an intellectual disability in these settings. Knowing which children have an intellectual disability can enable teachers to change and adapt their educational methods and approaches to create a learning environment that is appropriate and beneficial for these children (Committee on the Rights of Persons with Disabilities 2016).

Research indicates, however, that 14% of teachers in England felt they lacked the skills to identify children who were not progressing at the same rate as their peers (Department for Education 2020). Many teachers also have limited knowledge about intellectual disability and lack confidence in how best to identify and support these children (McKenzie et al. 2023; Rae et al. 2011).

This highlights the need for education staff to have a structured, evidence-based, and accurate means of identifying those children with a potential intellectual disability who may need further assessment and support. This has led to an increasing interest in the role of screening. Screening is not designed to replace diagnostic assessment, but rather aims to help identify those who may benefit from additional specialist assessment and intervention. Sapiets, Totsika, and Hastings (2021) highlight a need to explore the appropriateness and performance of developmental disability screening tools in different contexts, to enable staff to choose the best screening methods. This is echoed by Hutchinson (2021), who identifies the need for the ‘greater use of age-standardised assessments where appropriate instruments exist to increase consistency in assessment’ (p11).

Many developmental screening tools exist, but most are designed to screen for general developmental difficulties in children aged five years and younger (see Faruk et al. 2020; Sheldrick et al. 2020). Reviews of such tools also frequently exclude screening measures for specific conditions, such as intellectual disability (Cibralic et al. 2023; Komanchuk et al. 2023; Rah et al. 2023). There are, however, very few evidence-based tools available that are specifically designed to screen for intellectual disability and which are appropriate for children aged above five years (Nwokolo et al. 2023). The Hayes Ability Screening Index (Hayes 2000) screens for intellectual disability, but was developed for people aged 13 years and above, meaning that it would not be suitable for younger children. The Child and Adolescent Intellectual Disability Screening Questionnaire (CAIDS-Q) has been validated for children and young people aged six to under 18 years, making it suitable for school age children. It has been shown to accurately identify children with an intellectual disability in a range of service settings (e.g. McKenzie et al. 2012; McKenzie, Murray, and Murray 2013; McKenzie et al. 2019a) and where children have co-occurring autism (McKenzie, Metcalfe, and Murray 2023). It has not, however, been systematically evaluated when used in school contexts. The present study, therefore, aims to explore the performance and some of the psychometric properties of the CAIDS-Q when used in mainstream school settings.

## Materials and methods

### Ethics

The project received ethical approval from the Department of Psychology ethics committee of the first author’s university (Ref: 42813). All adult participants provided written consent to take part. The parents/guardians of the participating children provided written consent on their behalf.

### Design

A quantitative approach was used to explore the performance of the CAIDS-Q, including the agreement between the CAIDS-Q and clinical assessment of intellectual disability (sensitivity and specificity), convergent validity with scores on assessments of adaptive and intellectual functioning (see ‘Measures’ section below), as well as inter-rater reliability between parents and teachers when using the CAIDS-Q.

### Participants

The inclusion criteria for participants were that they were adults (aged 18 years or older); a teacher or parent/guardian of a child who was attending a participating mainstream school; had provided written consent; and who were able to complete the relevant measures about the children (for example, knew the children well enough and were sufficiently competent in the English language to complete the standardised measures). Overall, parents and/or teachers of 70 children took part. The inclusion criteria for the children were that their parent/guardian had consented to them taking part and they were attending a participating mainstream school. Parents and teachers were recruited *via* the schools that were participating in the project (see ‘Procedure’ below).

### Setting

The participating schools were all mainstream schools which were based in the North-East of England. In the UK, mainstream schools provide education to children with a wide range of abilities. They are contrasted with special schools which provide specialist support for children whose needs can’t be met by mainstream educational provision. A total of 19 schools were contacted and invited to participate. These schools were chosen to cover the full age range of school -aged children and to represent urban and rural areas in the North - East of England. Of the 19 schools which were contacted, 11 agreed to take part. Of these, seven were primary schools and four were secondary schools. Nine of the schools were located in urban areas. Of the eleven schools who agreed to take part, nine subsequently provided participants (five primary and four secondary schools).

### Measures

#### Assessment of intellectual disability

The three criteria for intellectual disability are: significant impairments in intellectual and adaptive functioning, and childhood onset (WHO 2022). These were assessed using the assessments below:

• Adaptive functioning: The Adaptive Behaviour Assessment Scales – Third edition (ABAS-3: Harrison and Oakland 2015). This is an age standardised assessment (covering people from 0 - 89 years) that includes some children with an intellectual disability in the standardisation samples. It measures adaptive skills identified as relevant to the criteria for intellectual disability (American Psychiatric Association 2013) which relate to three domains (conceptual, social, and practical). It also provides an overall General Adaptive Composite (GAC) score, which represents an overall summary of the person’s adaptive functioning. The ABAS has test-retest reliability and inter-rater reliability correlations of 0.74 and above. The ABAS has teacher and parent versions, and in the present study, both the parents and teachers were invited to complete the relevant version about the children. A GAC score of 70 or less was taken to indicate significant impairment of adaptive functioning.

• Intellectual assessment: The Wechsler Intelligence Scales for Children UK version– Fifth Edition (WISC V: Wechsler 2016). This is a commonly used assessment in the UK which is standardised for children and adolescents aged between six years and 16 years, 11 months. It has been found to have good psychometric properties, including internal consistency and test-retest reliability (see Bergeron et al. 2023). It provides five index scores and a full-scale IQ score (FSIQ). This assessment was administered on an individual basis, by an experienced clinical psychologist, with the child. For the purposes of the study, FSIQ was used to determine whether the child met the criteria of significant cognitive impairment (IQ of approximately 70 or less).

Screening measure: Child and Adolescent Intellectual Disability Screening Questionnaire (CAIDS-Q: McKenzie et al. 2012). This is a short measure with items relating to support needs, literacy, telling the time, friendships, contact with specialist services, and basic adaptive skills (see https://learningdisabilitymatters.co.uk/learning-disability-form/ for further details). The questions are presented in English and the seven core items have yes/no responses, which are converted to a percentage score (range 0-100%). The percentage score is compared with an age-adjusted cut-off score, as outlined in the scoring manual, and those who fall below the cut-off (43 and 71 for children aged below eight years and eight years and older respectively) are considered likely to meet the criteria for intellectual disability. The CAIDS-Q has previously been found to have sensitivity and specificity values ranging from 82-100% and 83-94% respectively, depending on the age of the child, the setting in which it was used, and whether the child was also autistic (e.g. McKenzie et al. 2012; McKenzie, Metcalfe, and Murray 2023; McKenzie, Murray, and Murray 2013; McKenzie et al. 2019a). In the current study, both the parents and teachers were invited to complete the CAIDS-Q online in relation to the children.

Information was also gathered online about the age and sex of the children, any existing condition/diagnosis, whether they spoke English as a second language and any existing support that they received at school.

### Procedure

Nineteen schools were emailed information about the study and invited to take part. They were also offered the opportunity to ask questions about the study. If a school agreed to take part, they were given information about the study to distribute to parents of children who attended the school. This information provided a link to an online platform where more detailed participant information was provided, including researcher contact details that the potential participants could use if they had any questions. All children at the participating schools were invited to take part and those parents who wished their child to participate could record their consent and complete the online CAIDS-Q and additional demographic information about the child. Parents were then asked to complete the ABAS about their child. The schools were informed about the children whose parents had consented and the relevant teachers were asked to complete the CAIDS-Q and ABAS in relation to them.

Any child who was indicated as having difficulties on either the CAIDS-Q, ABAS or both, on either the parent or teacher completed measures, was offered an intellectual assessment, usually within a month or less of the ABAS being completed. For the purposes of the study, ‘difficulties’ were defined as a CAIDS-Q score that was 43 or less for children under eight years and 71 or less for children aged eight years and above and/or an ABAS score that was in the ‘below average’ range or below (89 or less). Children who had an ABAS GAC score that was above the ‘below average’ range, by definition, did not meet the one of the three required criteria for intellectual disability (significant impairment in adaptive functioning) and, therefore, were not offered an intellectual assessment.

The clinical psychologist who completed the intellectual assessment was aware, at the point of completing the WISC that the children had difficulties as outlined above. Following the assessments of adaptive and cognitive functioning, feedback was provided to the parent and school about the outcome. In most cases, there was also liaison with the school educational psychologist in case additional support measures needed to be put in place for the child.

Children were allocated to one of two groups according to the outcome of the psychology assessment. Those who met the criteria for intellectual disability (Group 1) had an ABAS GAC score of 70 or less and a FSIQ of IQ of 69 or less. These scores were interpreted in the context of the error ranges of the scores and the overall profile of the child. Those who did not meet the criteria for intellectual disability (Group 2) had ABAS GAC and FSIQ scores above these values. A comparison was made between the classifications based on outcomes from the CAIDS-Q (i.e. whether the child was indicated as likely to meet the criteria for intellectual disability or not) and the outcome of the intellectual/adaptive functioning assessments (i.e. whether the child actually met the criteria for intellectual disability or not).

### Analyses

Convergent validity between CAIDS-Q, WISC V Full Scale IQ (FSIQ) and ABAS-3 General Adaptive Composite (GAC) scores was measured using Pearson’s correlation. FSIQ provides a summary indicator of cognitive functioning, while GAC provides a summary indicator of adaptive functioning. The effect sizes of the correlation coefficients were interpreted based on the guidance by Cohen (1992), as these values are argued to be more appropriate for behavioural sciences research (Hemphill 2003).

Fleiss’ Kappa was used to explore the agreement between teachers and parents on the CAIDS-Q outcome. The kappa coefficient was interpreted according to the guidance by Landis and Koch (1977).

Sensitivity and specificity values were calculated, using the formula outlined by Trevethan (2017). This compared the outcome of the CAIDS-Q with the clinical assessment of intellectual disability, i.e. the intellectual and adaptive functioning of the children. This is in line with recommendations that sensitivity and specificity values are based on gold standard or reference assessments (Trevethan 2017), rather than parent or teacher reports.

Sensitivity assesses the extent to which the CAIDS-Q correctly identified those children who were found on assessment to meet the criteria for intellectual disability. Specificity assesses the extent to which the CAIDS-Q correctly identified those children who were found on assessment not to meet the criteria for intellectual disability.

These values were calculated separately for assessments/screeners that were completed by the parents and those that were completed by the teacher. The resulting sensitivity and specificity of the CAIDS-Q was evaluated against the values recommended by researchers (e.g. Glascoe 2005) and professional bodies (e.g. American Academy of Pediatrics Committee on Children with Disabilities 2001) for developmental screening tools around 70% and above for sensitivity and 80% and above for specificity.

### Sample size

The required sample size was calculated, assuming an alpha value of 0.05 and a large effect size. The latter was based on the results of previous validation studies of the CAIDS-Q (e.g. McKenzie et al. 2012). A sample size of 50 was calculated as providing sufficient statistical power (at 0.80) for all the planned analyses (Cohen 1992).

## Results

### Demographic information

Assessments/screeners were completed in relation to 70 children. The parents completed 52 CAIDS-Q screeners and ABAS assessments about the children. Only one parent completed these about each child. The teachers completed 58 CAIDS-Q screeners and ABAS assessments (See Figure 1). Overall, 39 children had CAIDS-Q screeners completed by both parents and teachers and 40 had ABAS assessments completed by both. Table 1 provides the demographic information about the children and their CAIDS-Q, ABAS GAC and WISC V scores. Eight of the participating children were aged under six years.

**Figure 1.**
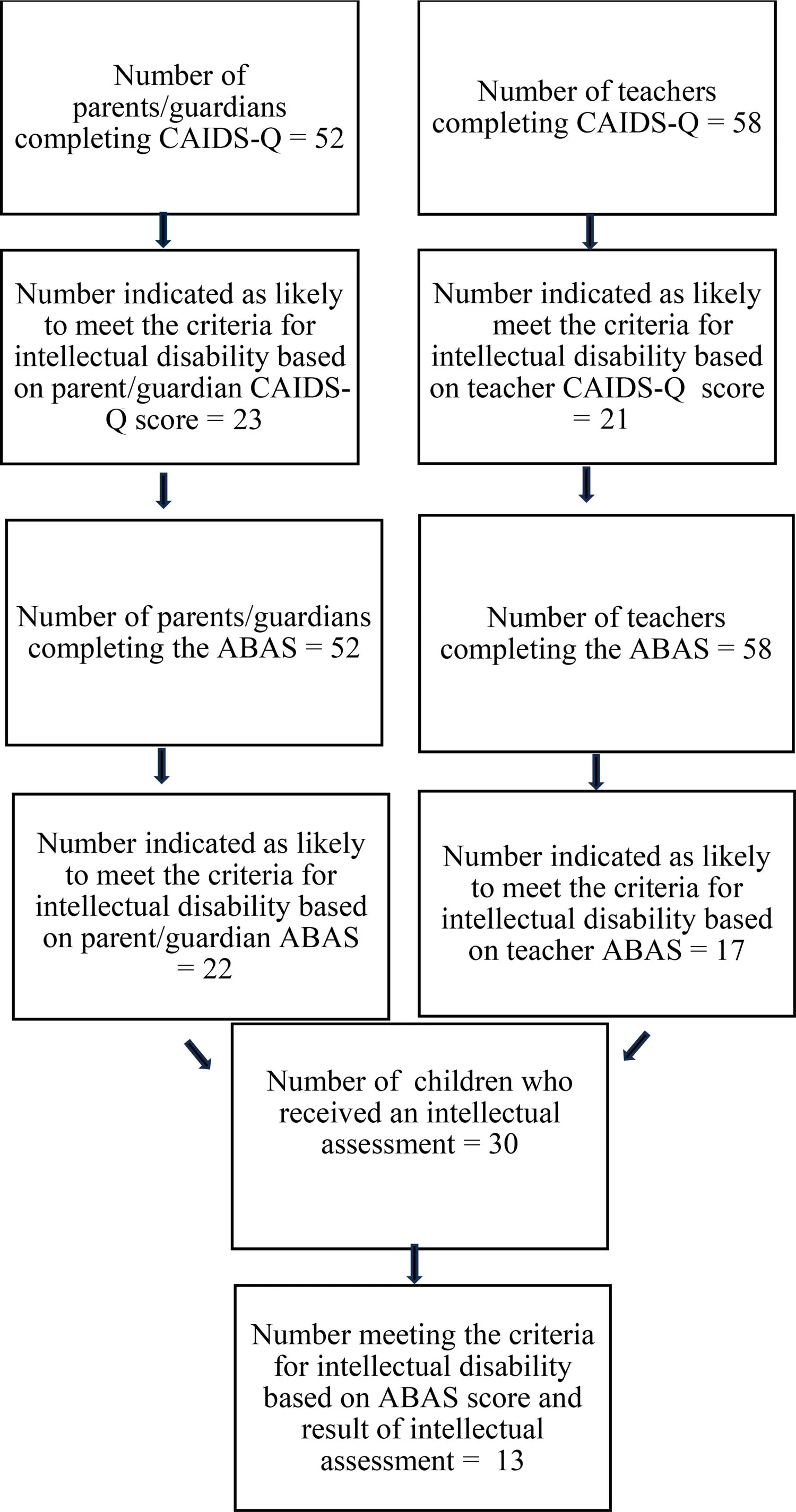
A summary of the outcomes based on scores from the CAIDS-Q, ABAS and intellectual assessment. *Note:* There is overlap in the two groups, with 39 children having had CAIDS-Q screeners completed by both parents and teachers and 40 having had ABAS assessments completed by both.

**Table 1.** Demographic information about the participants and their CAIDS-Q, ABAS GAC and WISC V scores.

	Total Sample (*n* = 70)			Parent completed measures (*n* = 52)		Teacher completed measures (*n* = 58)						
	Range	Mean (SD)		Range	Mean (SD)	Range		Mean (SD)				
CAIDS-Q score	14 - 100	62.7 (24.3)		0 - 100	54.4 (22)	14 − 100		61.3 (24.9)				
ABAS GAC score	48 - 112	82.5 (18.3)		47 - 105	74.1 (16.4)	48 - 112		83.4 (18.9)				
WISC V score	(*n* = 30)			(*n* = 26)		(*n* = 24)						
	49 - 105	80.2 (15.9)		49 - 105	78.9 (16.4)	49 - 105		80.1 (15.7)				
Age	4 years, 9 months − 15 years, 5 months	8 years, 9 months (2 years, 8 months)		5 years, 7 months − 15 years, 5 months	9 years (2 years, 9 months)	4 years, 9 months − 15 years, 5 months		8 years, 5 months (2 years, 6 months)				
	**Number (%)**											
Sex	**Male**	**Female**			**Male**	**Female**			**Male**		**Female**	
	41 (58.6)	29 (41.4)			31 (59.6)	21 (40.4)			32 (55.2)		26 (44.8)	
Race	**White**	**Black**	**Mixed**	**Other/ Did not specify**	**White**	**Black**	**Mixed**	**Other/ Did not specify**	**White**	**Black**	**Mixed**	**Other/ Did not specify**
	62 (89.9)	3 (4.3)	2 (2.9)	2 (2.9)	46 (88.5)	3 (5.8)	2 (3.8)	1 (1.9)	50 (87.7)	2 (3.5)	2 (3.5)	3 (5.3)
English spoken as a second language	**Yes**	**No**			**Yes**	**No**			**Yes**		**No**	
	12 (17.4)	57 (82.6)			10 (19.2)	42 (80.8)			8 (13.8)		50 (86.2)	

Table 2 shows the reported conditions and existing support at school for participants prior to screening and assessment.

**Table 2.** The reported conditions and existing support at school for participants prior to screening and assessment.

	Learning Difficulty	Intellectual Disability	Autism	None	Other
	**Number (percentage of participants)**				
Reported condition^a^ (*n* = 67)	13 (18.6)	6 (8.6)	6 (8.6)	35 (50)	14 (20)
	**Yes**	**No**	**Unknown**		
Currently receiving support at school (*n* = 68)	34 (50)	33 (48.5)	1 (1.5)		

^a^Note some individuals reported more than one condition.

### Sensitivity and specificity values for the CAIDS-Q

Table 3 shows the sensitivity and specificity values for the CAIDS-Q. These results are presented separately for the parent completed assessments/screeners and teacher completed assessments/screeners.

**Table 3.** The sensitivity and specificity values for the CAIDS-Q for the parent and teacher completed assessments.

	Sensitivity	Specificity
Parent/guardian	92%	70%
Teacher	100%	79%

### False positive outcomes on the CAIDS-Q

Ten children (male = 5, female = 5, age range = 5 years, 5 months to 12 years, 2 months) were incorrectly identified by their teacher CAIDS-Q score as likely to have an intellectual disability, when subsequent assessment showed they did not meet the criteria for intellectual disability (false positives). Two children, both males, were identified as false positives by their parent CAIDS-Q scores, one of whom was also identified as a false positive by the teacher scores. The two children were aged 10 years, 4 months and 10 years, 5 months respectively. An examination of the GAC scores for the 12 children found that seven had adaptive functioning in the significant impairment range (70 or less), two had scores in the low range (71-79) and two had scores in the below average range (80-89). In respect of their intellectual functioning, six of the twelve children had WISC V scores in the low average range. A WISC V score was not available for one child. Figure 2 illustrates the relationship between the GAC scores and FSIQ scores for the remaining 11 children.

**Figure 2.**
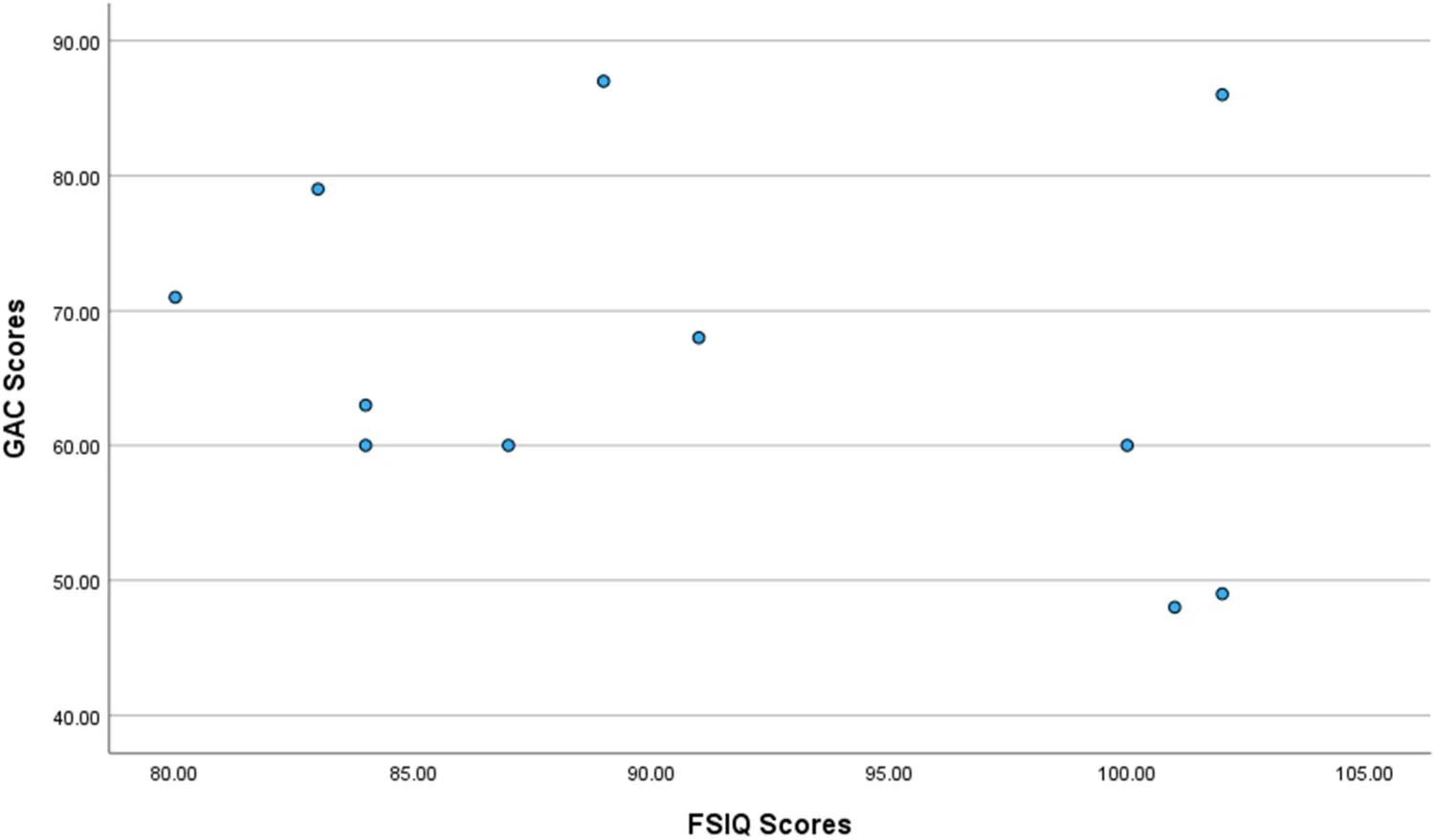
The relationship between the GAC scores and FSIQ scores for children who were ‘false positives’ on the CAIDS-Q. *Note.* A WISC score was not available for one child.

### Inter-rater reliability

Fleiss’ kappa was used to explore the inter-rater agreement between teachers and parents on the outcome of the CAIDS-Q (likely or unlikely to meet the criteria for intellectual disability). There was agreement between teachers and parents for 12/18 children (67%) who were classified by the CAIDS-Q as likely to have an intellectual disability and for 18/21 children (86%) of those classified as unlikely to have an intellectual disability. The result indicated moderate agreement between the raters (κ = 0.527 [95% CI, 0.213 to 0.841], *p* < 0.001).

### Convergent validity

The correlations between CAIDS-Q scores, ABAS GAC scores, and WISC V Full Scale IQ (FSIQ) scores and their associated effect sizes are shown in Table 4.

**Table 4.** Correlations between CAIDS-Q, WISC V FSIQ, and ABAS GAC scores and the associated effect sizes.

	WISC FSIQ		ABAS GAC score	
	R value	Effect Size^a^	R value	Effect size^a^
CAIDS-Q (Parent/guardian)	.658*	Large	.376**	Medium
CAIDS-Q (Teachers)	.713*	Large	.374*	Medium

*p is significant at 0.01 level (one-tailed), **p is significant at 0.05 level (one-tailed).

^a^Based on Cohen’s guidelines (1992).

#### Identification of those with an intellectual disability

Thirteen children were indicated by the CAIDS-Q as likely to meet the criteria for intellectual disability and subsequent assessment confirmed this. Of these, only three were previously reported by their teacher to have an intellectual disability, although twelve of the children were reported by teachers as already receiving some form of additional support at school.

## Discussion

The study had a specific focus on the use of the CAIDS-Q in mainstream schools because few evidence-based screening tools exist for school-aged children that are specific to intellectual disability (Nwokolo et al. 2023). The aim of the study was to explore some of the psychometric properties of the CAIDS-Q when used in mainstream schools. In respect of convergent validity, the CAIDS-Q scores for the parent and teacher data had significant, positive correlations with the ABAS and WISC scores, with medium and large effect sizes respectively. This pattern of results is consistent with those found in previous validation studies of the CAIDS-Q in health service settings (McKenzie et al. 2012, 2013; McKenzie et al. 2019a).

Moderate agreement was found between teachers and parents in respect of the outcome of the CAIDS-Q, i.e. whether the score indicated that the child was likely to meet the criteria for intellectual disability or not. Differences between parent and teacher responses on the CAIDS-Q may reflect the different settings in which they observe the abilities of the child. Emam et al. (2020) note that teachers are less likely to be able to comment on the child’s skills in relation to practical domains such as self-care or home living, but more likely to be able to observe conceptual skills, such as reading and writing, and social skills, with the opposite being true for parents.

The results also indicated that the sensitivity value of the CAIDS-Q when completed by teachers was greater than the minimum recommended levels for developmental screening tools (American Academy of Pediatrics Committee on Children with Disabilities 2001; Glascoe 2005). In this study, the sensitivity value illustrates the ability of the CAIDS-Q to accurately identify children with an intellectual disability, as compared with the outcome from a gold standard assessment of intellectual disability. The value of 100% for the teacher data, shows that the CAIDS-Q correctly identified all of the children who were assessed as meeting the criteria for intellectual disability.

The specificity value illustrates the ability of the CAIDS-Q to accurately identify those who do not have an intellectual disability based on assessment. The value of 79% for the teacher data shows that the CAIDS-Q correctly identified 37 out of the 47 children who did not have an intellectual disability. These values are also similar to those found when using the CAIDS-Q in health and other settings (e.g. McKenzie et al. 2012; McKenzie, Murray, and Murray 2013; McKenzie et al. 2019a) or with children with co-occurring autism (McKenzie, Metcalfe, and Murray 2023).

### Implications for practice and policy

Previous research has indicated that there is a need for accurate, age-appropriate, evidence-based screening tools (Hutchinson 2021; Sapiets, Totsika, and Hastings 2021). This is particularly important in the context of criticisms of existing systems of identifying children with additional support needs (Hutchinson 2021), and research suggesting that many teachers lack the knowledge, skills, training, and confidence to identify and support these children (Department for Education 2020) and specifically, children with intellectual disability (McKenzie et al. 2023; Rae et al. 2011).

It is doubtful that any developmental screening tool will be 100% accurate (Rah et al. 2023) as a balance needs to be struck between sensitivity and specificity. The current results suggest that the CAIDS-Q is accurate at identifying children who have an intellectual disability in mainstream school settings. In addition, all but one of the children who were false positives, i.e. wrongly classified by the CAIDS-Q as likely to meet the criteria for intellectual disability, were found, on assessment, to have some degree of difficulty with either their day-to-day skills, intellectual functioning, or both. This suggests that the CAIDS-Q may also have a role in highlighting children who would not meet the criteria for intellectual disability, but who may still benefit from further assessment and support because of challenges with their cognitive and adaptive skills. In this context, previous research has indicated that the CAIDS-Q can help validate the concerns of parents and professionals and give them confidence when trying to obtain further assessment or additional support for a child (McKenzie et al. 2019b; McKenzie et al. 2023). Due to the small number of children who were false positives, it was not possible to determine if there was a particular pattern to the results. Future research, with a larger sample size, could help address this question.

While the sensitivity and specificity values of the CAIDS-Q were at or near the recommended minimum values for developmental screening tools for both parents and teachers, the results were more accurate when completed by teachers. This may be because the ABAS assessment of adaptive skills has parent and teacher versions of the questionnaires that measure adaptive skills in slightly different contexts. As a result, the teacher questionnaire may more closely correlate with intellectual skills because of the greater focus on academic skills. Previous research suggests that, while there is usually a strong association between adaptive and intellectual skills, this relationship is not perfect and that further research in this area is needed in respect of people with an intellectual disability (e.g. Mattie et al. 2023).

If screening for intellectual disability were to be introduced more widely in mainstream schools, there would need to be some consideration of when and with whom the CAIDS-Q should be used. It is recommended that assessment of intellectual disability is repeated during childhood, as the age and development of the child can affect the outcome (World Health Organization 2022). As the same factors would influence the outcomes of the CAIDS-Q, it is recommended that screening should also be carried out at different ages, with stakeholders suggesting that this should take place when the child is approximately age 6 years old and at important transition points, such as the final year at primary school or first year at secondary school (McKenzie et al. 2023).

It would also need to be considered whether it is more beneficial to offer screening to all children or only those about whom there are already concerns. A longstanding criterion that is helpful when considering the introduction of a screening programme is whether it will provide more benefits than disadvantages (Wilson and Jungner 1968). While offering screening to all children at an early stage in their school journey may require more time and resources, and may result in some over-identification, it would help ensure that children with difficulties were not missed (McKenzie et al. 2023) and that access to appropriate support and services was improved. While there has been limited direct research into the relative costs and benefits of over-identification, versus under-identification of intellectual disability, it has been suggested that the financial costs of the latter are greater than the former, when considering the wider category of developmental disabilities (Barnett and Escobar 1990).

In addition to financial considerations, early identification can also bring benefits for children and family members in terms of increased understanding, earlier intervention and support with daily skills, cognitive functioning, and behavioural challenges (see Guralnick 2017; McKenzie et al. 2021; Sapiets, Totsika, and Hastings 2021). In the present study, only three of the children who were assessed as meeting the criteria for intellectual disability, were reported by teachers as already being identified as such. While 12 of the 13 children were already receiving some support at school, research suggests that the use of the CAIDS-Q can lead to quicker identification, accessing extra or different forms of support for the child and wider family, and improved understanding and acceptance of the child and their needs (see McKenzie et al. 2019b; McKenzie et al. 2021). Identifying that a child has an intellectual disability can also provide access to additional and tailored health provision, including ‘reasonable adjustments’ in their health care, such as longer appointment times and the provision of accessible information.

Taken together, the available evidence suggests that screening all children, but only at key points in their school journey would offer the best balance between costs and benefits. Future research which followed up those who were identified by screening as likely to have an intellectual disability would help clarify the longer-term impact of screening and early identification in schools on both educational and health outcomes.

There would also need to be consideration of the context within which the CAIDS-Q can best be used. It was developed to be widely accessible and does not require the person using it to hold a particular professional qualification or to need training in its use (McKenzie et al. 2012). The online version, which is available at the Learning Disability Matters for Families website (https://learningdisabilitymatters.co.uk/tools/) provides immediate feedback to the user, based on their responses to the questions, about whether the child is likely to meet the criteria for intellectual disability or not. Links are also provided within the feedback section and wider website to further sources of support and information. Depending on the area that the user lives in, there are also links to local sources of support. While training for using the CAIDS-Q is not required, it may be useful to signpost teachers who intend to use it to broader awareness training about intellectual disability (e.g. Health Education England 2023), as research suggests that some lack knowledge and confidence in this area (McKenzie et al. 2023; Rae et al. 2011).

The CAIDS-Q could also be implemented as part of existing educational frameworks that are designed to support children with additional needs. In the UK, for example, the SEND Code of Practice outlines the ‘Assess, Plan, Do, Review’ framework (Department for Education and Department of Health 2015). This aims to help schools to develop tailored support strategies for children with additional needs, based on information that is received from different stakeholders and assessments. Following implementation, the effectiveness of the strategies is evaluated (Davies and Henderson 2020). The CAIDS-Q could offer an important source of information at the ‘Assess’ stage of this framework.

### Limitations

The study had some limitations, all of which may influence the generalisability of the results. The sample size was relatively small and was based on nine participating schools, predominantly in urban areas, from one region of the UK. These schools may have taken part because of a particular interest in intellectual disability. In addition, the mean ABAS GAC scores fall below the average range for both parent and teacher completed measures. This suggests that parents may have been more likely to participate if there were some concerns about their child. While the results are broadly consistent with those found in other service settings and geographical areas (McKenzie et al. 2012; McKenzie, Murray, and Murray 2013; McKenzie et al. 2019a), there should be some caution about the extent to which the results can be generalised more widely. Further research with a larger number of participants from more schools across different regions and with a more equal balance of schools located in urban and rural areas would increase the diversity of the sample and help to confirm if the findings can be applied more widely. As many schools in the UK are organised into educational trusts which comprise of multiple schools, recruiting *via* such trusts in multiple regions offers a way to increase the diversity and size of the sample. In addition, the results of the present study may offer an illustration to potential participants of the benefits of using the CAIDS-Q, which may increase recruitment to future studies.

A second consideration was that the inclusion criteria resulted in a small number of children aged under six years taking part in the study because they were attending a participating school. The youngest age that the CAIDS-Q has previously been validated with is six years (McKenzie, Murray, and Murray 2013). It can be more difficult to accurately identify mild intellectual disability in younger children because of developmental changes in the context of changing environmental demands. It is suggested that screening (McKenzie and Murray 2022) and clinical assessment (WHO 2022) should be repeated throughout the developmental period to address this. All but one of the eight children who were aged under six years in the present study were, however, correctly classified by the CAIDS-Q. A larger study that specifically validated the CAIDS-Q with children aged under six years would be helpful to ensure that it has the same standard of psychometric properties with this group as it does for children aged six years and older.

A third limitation was that there were likely to be differences in how long the teachers had taught the participating children. While one inclusion criterion for the study was that respondents felt able to complete the study measures, teachers who were less familiar with some children may have given less accurate responses. Future research could explore if there is an optimal time for teachers to complete the CAIDS-Q, to take account of how well they know the children at different points in the school year.

Finally, only children meeting certain criteria were offered an intellectual assessment. As a result, the psychologist who conducted the intellectual assessments was aware that the children who were offered a WISC assessment had some difficulties with their adaptive functioning as indicated by their scores on the ABAS and/or CAIDS-Q. This did not, however, indicate whether the child’s performance fell within a range that indicated likely intellectual disability or not, as children with scores above this range, but below the ‘average’ range were invited to complete the intellectual assessment. Future research could offer intellectual assessments to all participating children.

## Conclusion

The results of the study indicate that the CAIDS-Q, when used in mainstream schools, had psychometric properties that were consistent with those found in health settings. The routine use of evidence-based intellectual disability screening tools may help to ensure that children with intellectual disability are identified and supported from as early an age as possible.

## Data Availability

Data are available from the first author on reasonable request.
